# When giants talk; robotic dialog during thoracolumbar and sacral surgery

**DOI:** 10.1186/s12893-022-01546-7

**Published:** 2022-04-01

**Authors:** Josh E. Schroeder, Saadit Houri, Yoram A. Weil, Meir Liebergall, Rami Moshioff, Leon Kaplan

**Affiliations:** Orthopedic Complex, Hadasash Hebrew University Medical Center, Kiryat Hadassah, POB 12000, Jerusalem, Israel

**Keywords:** Robotic guidance, Intraoperative imaging, Pedicle screw accuracy, Robotic spine surgery, Hybrid operating room, 3D fluoroscopic imaging

## Abstract

**Background:**

Spinal trauma patients treated in a specialized hybrid operating room (OR) using two robotic systems communicating during surgery.

**Methods:**

Retrospective review of patients with thoracolumbar or sacral fractures who underwent surgical fixation between Jan 2017 to Jan 2020 with robotic-guided percutaneous pedicle screw insertion in the specialized hybrid OR with Robotic flat panel 3D C-arm (ArtisZeego) for intraoperative interventional imaging connected with the robotic-guidance platform Renaissance (Mazor Robotics).

**Results:**

Twenty eight surgeries were performed in 27 patients; 23 with traumatic spinal fractures, 4 with multi-level thoracolumbar compression fractures due to severe osteoporosis. Average patient age 49 (range 12–86). Average radiation exposure time 40 s (range 12–114 s). Average radiation exposure dose 11,584 ± SD uGym^2^ (range 4454–58,959). Lumber levels operated on were between T5 and S2 (shortest three vertebras and longest eight vertebras). 235 (range 5–11) trajectories were performed. All trajectories were accurate in all cases percutaneous pedicle screws placement was correct, without breach noted at the pedicle in any of the cases. No major complications reported. In all cases, follow-up X-rays showed adequate fracture reduction with restoration.

**Conclusions:**

Merging of surgical robotics technologies increases patient safety and surgeon and patient confidence in percutaneous spine traumatic procedures.

## Background

Over the last two decades, thoracolumbar and sacral fractures due to high-energy trauma are commonly treated by minimally invasive surgical techniques. To establish spinal stabilization the insertion of percutaneous pedicle screws is routinely performed in cases that do not require decompression [1].

New technological developments over the last three decades have allowed for increased safety and accuracy in percutaneous pedicle screw fixation procedures in the lumber spine [2–6]. These include several different navigational techniques, as fluoroscopy-assisted, computed tomographic (CT)-guided navigation, and robotic-assisted navigation. Moreover these newer systems were found to reduce the incidence of screw misplacement and pedicle breaching associated with risk of neurologic and vascular compromise [7–10].

Intraoperative movements of the target anatomy, mainly due to manipulations or fragment motion are potentially limiting factors for accurate percutaneous pedicle screw placement, even in robotic-assisted spinal surgeries. Previous solutions proposed include the use of specific external intraoperative fixation devices, [11] or by using intraoperative imaging modalities to increase accuracy of the positioning during surgery [10, 12].

We aimed to find a solution by connecting two robotic systems—a robotic 3D C arm—for intraoperative interventional imaging with the robotic guidance platform Renaissance (Mazor Robotics Ltd., Caesarea, Israel) [13], to enable real-time intraoperative post positioning and manipulation imaging, instrument and anatomy tracking, while still using the pre-planned, robotically guided navigation for proper positioning of the pedicle screws, as well as accurate verification of performance. In this study we present the first cohort of spinal trauma patients treated in a specialized hybrid operating room (OR) with the use of two robotic systems that communicate during surgery.

## Materials and methods

### Patient population

A retrospective review was conducted of the charts of all patients presented to a tertiary trauma center between Jan 2017 to Jan 2020 with thoraco-lumbar or sacral fractures who underwent surgical fixation without decompression (AO fracture subtype A or B without neurological deficits) with robotic-guided percutaneous pedicle screw insertion in the specialized hybrid OR with the two connected robotic systems. Informed consent was obtained from the patients before the surgical procedures were performed. The local institutional review board approved the protocol for this retrospective study. Any patient that needed a decompression was excluded from the study.

Data collection included indication for surgery, fracture pattern, patient’s sex, age; radiation exposure time and dose; number and accuracy of trajectories, number of levels operated on, duration of surgical procedure and post-operative complications. Patients were followed for 12 months after the surgery.

### Robotic systems

In the specialized hybrid OR, the following two robotic systems were connected for 3D fluoroscopic imaging with robotic guidance navigation:

Robotic flat panel 3D C-arm (ArtisZeego, Siemens AG, Forchheim, Germany), a robotic C-arm capable of large volume, high resolution 3D fluoroscopic scans, a multi-axis system for interventional imaging [14]. The main advantage of this system is in accurate repetitive intraoperative—post positioning and manipulation imaging, thus enabling the surgeon to keep his operative environment uninterrupted (i.e. the robot comes and go as needed).

Renaissance (Mazor Robotics Ltd., Caesarea, Israel) is a bone-mounted miniature robotic guidance system (9 cm tall, 5 cm diameter; 400 g), clinically tested for spinal surgery, featuring a six degrees of freedom Stewart–Gough platform allowing proper trajectory planning and execution [13]. The Renaissance robot is connected to a workstation, which runs specially designed graphic user interface software that allows real-time motion control.

In the classic setting of the Renaissance, the surgical planning is done on a 3D model of the patient's spine generated by the system, based on a preoperative CT scan. As the operation begins, a platform is directly attached to the patient's bony anatomy. A specialized 3D array is placed on top of the platform and two fluoroscopic images are taken (one in AP and one 60^0^ oblique) to allow the system to perform an automatic merging of the fluoroscopic images with the preoperative CT. This registration process connects the actual location of the patient and the platform on which the miniature robotic unit is placed with the preoperative CT and the surgical plan that it contains. Once this process is completed, the robotic unit can be dispatched to any of the planned trajectories and the surgical work begins, with the surgeon instrumenting through a cannula that is mechanically guided by the robotic unit.

### Robotic procedure in the hybrid OR

The patients underwent regular preoperative management: a general anesthetic induction, endotracheal intubation, preoperative antibiotics and neuro-monitoring. All patients were operated on lying in the prone position on the Aris Zeego table, the position of the head was towards the Artis Zeego imaging C-arm in order to allow full imaging rotation.

**Fig. 1:**
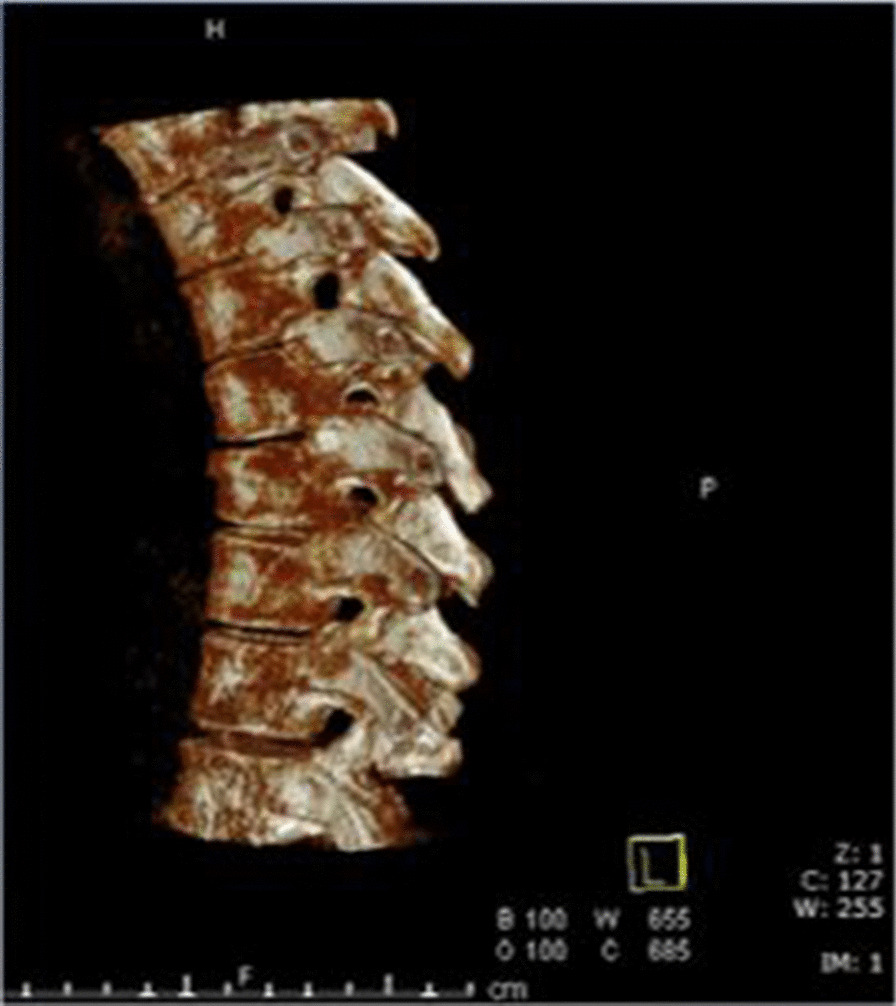
A 3D-flouroscopic scan performed by the Artis Zeego imaging robot

**Fig. 2:**
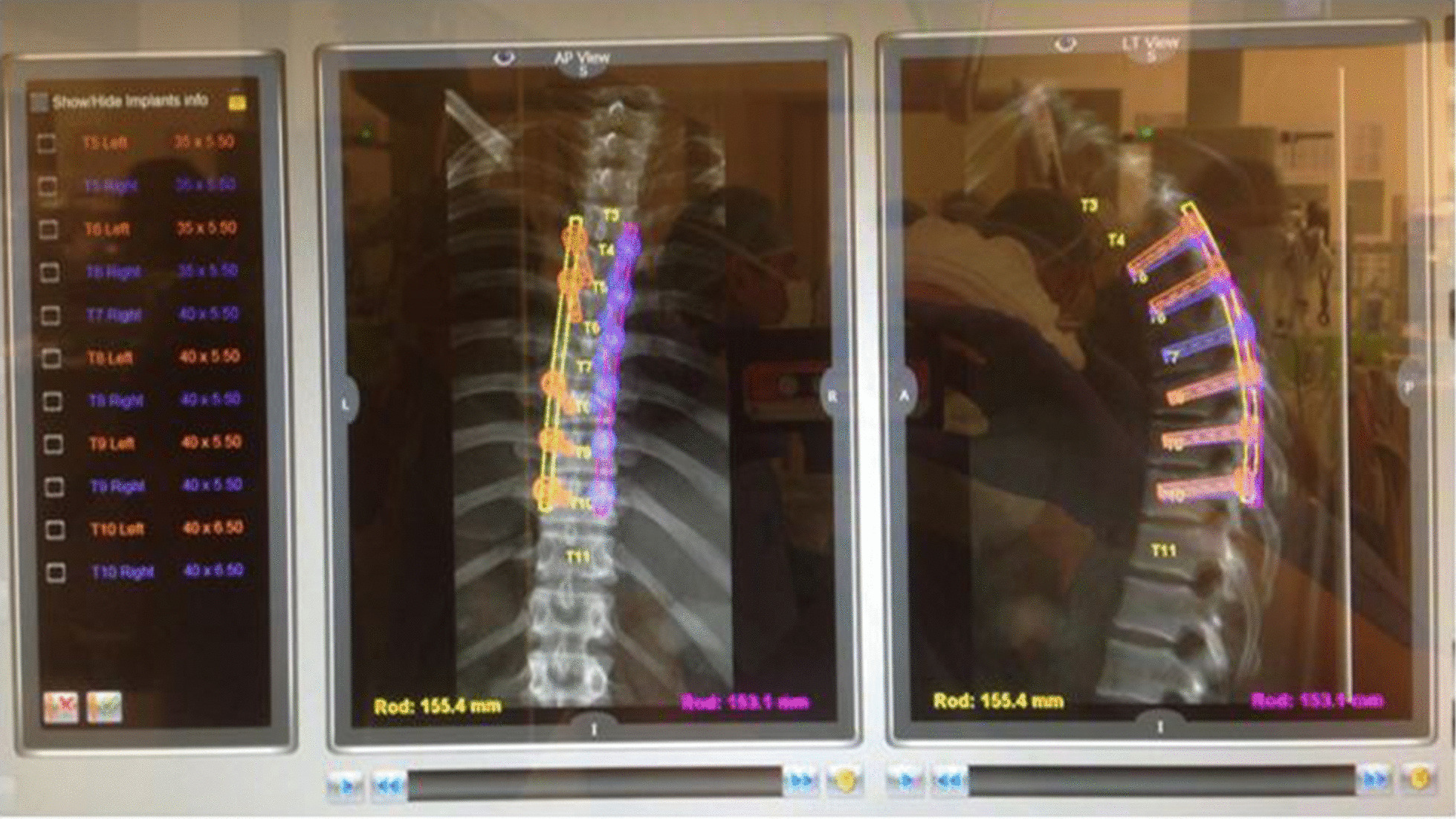
Using the Renaissance robotic system’s proprietary software the vertebras were identified and segmented; screw trajectories were planned in accordance to vertebral anatomy

In the hybrid OR after patient positioning and\or fracture manipulation a robotic star marker was placed over the designated part of the spine. A 3D-flouroscopic scan was performed by the Artis Zeego imaging robot (Fig. 1) and the DICOM images were transformed to the surgical guidance Renaissance robotic station. The vertebras were identified and segmented using the Renaissance robotic system’s proprietary software. Screw trajectories were planned in accordance to vertebral anatomy (Fig. 2).

**Fig. 3:**
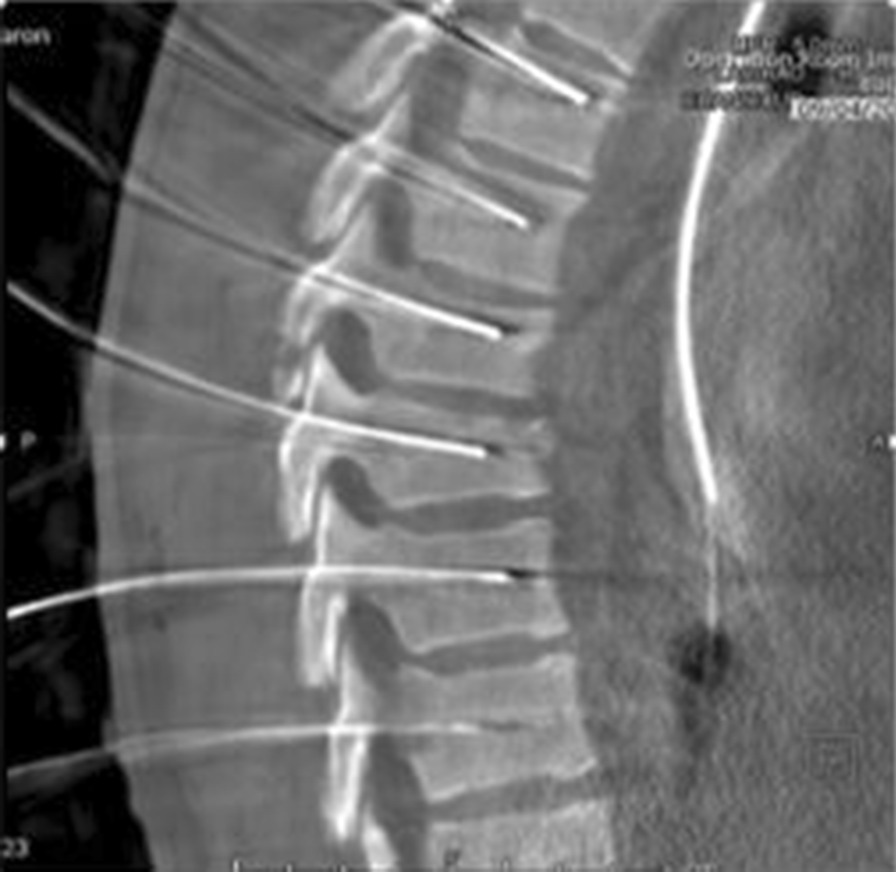
Using the Artis Zeego imaging robot another 3D-fluoroscopic scan verified trajectories

Trajectories were executed according to the individual plan of each patient and were verified with another 3D-fluoroscopic scan by the ArtisZeego table (Fig. 3). After validation of the exact placement of all trajectories and neuro-monitoring signals, hardware placement was performed in the routine manner.

In order for this dialog to occur, several optimizations of both robots were needed. Data output had to be changed, and settings on the imaging robot scanner needed to be modified for complying with the guidance navigation assistance robot requirements. Additionally, as the guidance robot utilizes software that allows planning multiple segments in one scan, the imaging robot capabilities were stretched to their fullest with larger 'sutured' scans being tested for compatibility with the guidance robot.

A post insertion scan was performed to make sure all screws were placed in the planed position.

## Results

### Descriptive data

**Fig. 4:**
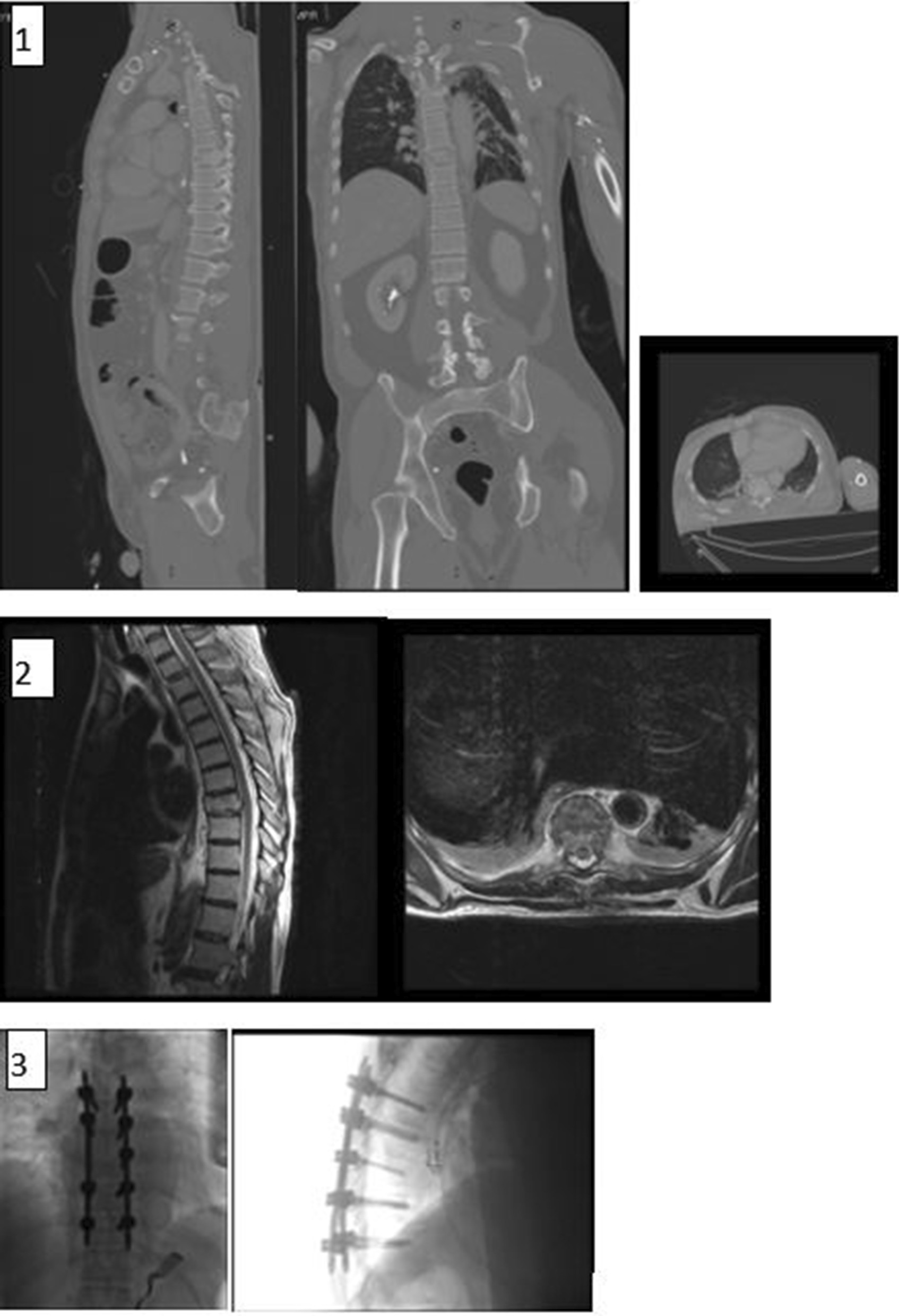
Case 1: 65 Y/O male was admitted with (**1**) T8 burst fracture due to electrical bicycle accident. (**2**) The MRI showed no cord injury. (**3**) The patient had a percutaneous fixation T6-T10 using robotic guidance and 3D intraoperative imaging. The patient had no complications

**Fig. 5:**
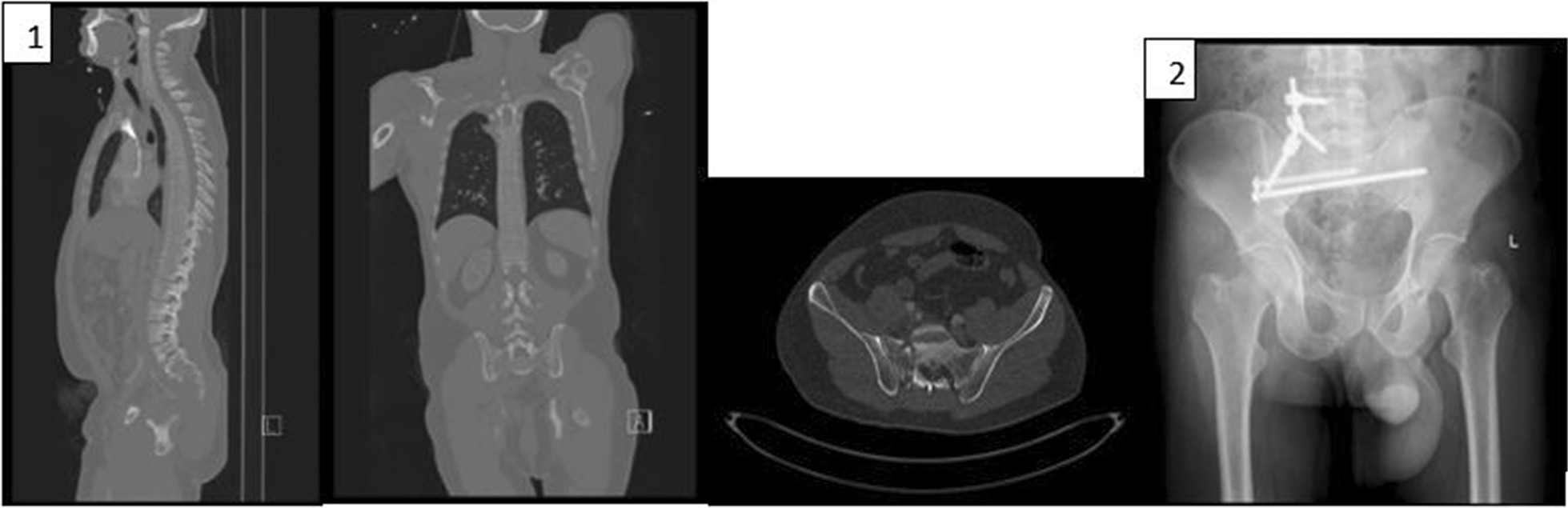
Case 2: 39 Y/O male was admitted with (**1**) Vertical Sheer fracture after fall from height. (**2**) The patient had a percutaneous fixation L4-S1 using navigation and 3D intraoperative imaging. The patient had no complications

**Fig. 6:**
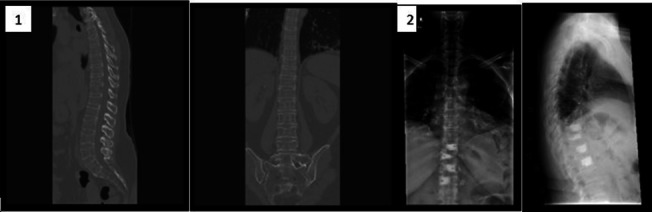
Case 3: 48 Y/O female with Bechet disease was admitted with (**1**) multilevel osteoporotic FX due to prolonged steroid use. (**2**) The patient had a percutaneous augmentation L4*2, L3, T9-12 using robotic guidance and 3D intraoperative imaging. The patient had minimal cement emboli after the procedure

Twenty-eight surgeries were performed in 27 patients, 16 male and 12 female, during the study period. 23 patients presented with traumatic spinal fractures, diagnosed with burst, flexion distraction, spino-pelvic instability or extension types of fractures (Table 1; see two example cases in Figs. 4, 5) and four patients were diagnosed with multi-level thoracolumbar compression fractures due to severe osteoporosis (see example case in Fig. 6). Average patient age was 49.1 ± SD (range 12–86). Average radiation exposure time was 41 ± SD seconds for the entire cohort (range 12–114 s). Average radiation exposure dose was 11,584 ± SD uGym^2^ (range 4454–58,959). A total of 235 (range 5–11) trajectories were performed. Lumber levels operated on were between T5 and S2 (shortest three vertebras and longest six vertebras). Mean surgical time was 276 ± SD minutes (range 75–415 min).

**Table 1:** Fracture types

Fracture type	Number of patients
Multiple compression fracture	4
Burst fracture	12
Flexion distraction (Chance fracture)	6
Extension fractures	4
Spino-pelvis instability	2

### Outcome data

All trajectories were accurate (235/235) and in all cases percutaneous pedicle screws placement was correct, without any breach noted at the pedicle in any of the cases. All patients were discharged without any major complication. There was one minor complication in one patient, a local wound infection, treated with oral antibiotics. In all cases, follow-up X-rays showed adequate fracture reduction with restoration.

## Discussion

In this study two robots were used to collaborate during thoracolumbar or sacral fracture surgical fixation. The first an imaging robotic 3D C-arm (ArtisZeego) that allows accurate intraoperative imaging and the second the Renaissance robotic navigation platform (Mazor Robotics) that assists in proper pedicle screw placement based on intraoperative image acquisition.

The study period implementation of the combined usage of these technologies was prior to the development of the newer robotic navigation platforms, when these technologies had yet to be combined as such. We found that the dialog created between these two robotic technologies increases patient safety and improves surgical results. The ability to have repeated real time intraoperative imagining in traumatic spine cases allowed us to increase the spectrum of cases that can be treated percutaneously, with improved robotic guidance for screw placement and validated instrumentation positioning.

The accuracy of percutaneous pedicle screw placement has been extensively studied and compared between the various technologies. Using the freehand technique the reported malposition rate is 10–15% of cases [4, 15]. Indeed, compared to freehand, the use of computer assisted navigation has achieved improved accuracy by 11% as first reported in a large meta-analysis, [4] additional meta-analyses further confirmed the superior accuracy of the robotic navigation technology [3, 6, 16].

Prior to combing these two technologies, in our previous robotic assisted surgery cases the percutaneous pedicle screw malposition rate was 5% (data collected in our institution). Others reports using the Renaissance (Mazor Robotics) reported similar rates [12, 17–21]. Keric et al. reported in the placement of 2067 screws in 406 patients an accuracy rate of 96.9% [17]. A growing body of evidence in the literature reports on high rates of pedicle screw positioning accuracy associated with low complication rates with the newer systems as 3D fluoroscopy and intraoperative CT integrated with navigational systems and robotic assisted navigation [12, 18]. Hyun et al. reported superiority in accuracy of placement of 130 screws in the 30 patient Robotic assisted group without a single misplaced screw when compared to the free hand technique with only 1.44% misplaced screws [18]. Similarly, Laudato et al. reported in 84 patients placement of 569 screws, no significant difference and very low rates of screw misplacement, between Robotic assisted (1.56%), O-arm (2.62%) and free hand technique (2.55%), which they attribute to the surgeon’s experience [12].

While in this series, we have shown an increased rate to 100%—achieving ultimate patient safety and accuracy. Although trajectory drilling and hardware placement were executed manually, these surgeries are another step towards surgical automation. This high rate of accuracy was achieved be real time imaging of the screws—as the patient was placed in the prone position in a fixed bed—the ability of the imaging of the spine in this position decreased vertebrae motion, in addition if there were any questions on fragment motion, a repeated scan was performed to maximize accuracy.

These findings have been since validated in studies using the newer Mazor X robotic guidance platform, which has an option of combination with intraoperative CT-based spinal navigation as reported by Khan et al. in 50 patients with degenerative disc disease who underwent robot guided placement of 190 pedicle screws achieving an accuracy rate of 99.5% [22]. Recently, initial results from a high-power multi-center prospective study from the U.S. MIS-ReFRESH compared 374 Mazor robotic-guided to 111 fluoroscopic guided minimally invasive spinal fusions and found Mazor robotic-guided had 5.2 fold lower risk of surgical complications and 8.8 fold lower risk for revision surgery [23]. While a similar multi-center prospective comparative European Robotic Spinal Instrumentation study (EUROSPIN) is still ongoing [24].

Operating room time is a further consideration regarding this method. Several authors described the surgeons quick learning curve of the new spinal navigation techniques for robotic or imaged guided pedicle screw placement and thus decreased surgical time per screw [10, 19, 25, 26]*.* In our study all cases were performed by surgeons experienced with this technique, thus allowing quick screw placement. The learning curve was mainly for proper patient positioning in the hybrid OR and for proper draping and bridge placement to allow the Artis Zeego fixed imaging device to perform a clean spin of the patient's spine. Once this challenge was overcome, case time dropped with cases taking as short as 75 min. Richter et al. presented similar challenges using an Artis Zeego hybrid OR in combination with a robotic navigation, yet concluding no considerable time was added to each procedure [27].

In hybrid ORs the use of a fixed imaging device (as the 3D robotic C-arm used in our study) are associated with risk of radiation exposure and pose a significant occupational hazard for surgical staff. A recent report recommended leaving the OR as no radiation during a 3D scan was measured behind closed doors of the OR [28]. Correspondingly, according to our Artis Zeego imaging protocol all OR staff are in a protected room while the imaging takes place, thus decreasing radiation exposure to a minimum. Furthermore, due to modifications in the Artis Zeego software, which automatically adapts the radiation dosage to the anatomic region, while assuring good constant image quality, the reduced dosage protocol in the thoracolumbar spine further reduces patient radiation exposure, as compared to a conventional post-operative CT scan [14]. As we have also previously demonstrated, robotic surgery has been shown to reduce radiation exposure [29].

There are several challenges in this technique; first, these procedures require designated rooms with a staff that can position these patients in a way to allow the scan to happen without any collisions. Secondly, as the patients head is covered anesthesia introversions may contamination of the surgical field. Finally and change in patient position requires a new scan—increasing patient radiation exposure.

The limitations of this study are its lack of randomization. However, this new merging of technologies is another step towards surgery automation decreasing malposition and increasing patient safety.

## Conclusion

The combination of surgical robots increases patient safety in percutaneous spine traumatic procedures. We believe this merging of technologies is a small step forward benefiting with our patients.

## Data Availability

The datasets generated during and/or analyzed during the current study are available from the corresponding author on reasonable request.

## Abbreviations

CT: Computer tomography
OR: Operating room
3D: Three dimensional
